# The Carotid Intima-Media Thickness and Arterial Stiffness of Pediatric Mucopolysaccharidosis Patients Are Increased Compared to Both Pediatric and Adult Controls

**DOI:** 10.3390/ijms18030637

**Published:** 2017-03-15

**Authors:** Raymond Y. Wang, Kyle D. Rudser, Donald R. Dengel, Elizabeth A. Braunlin, Julia Steinberger, David R. Jacobs, Alan R. Sinaiko, Aaron S. Kelly

**Affiliations:** 1 Division of Metabolic Disorders, CHOC Children’s Specialists, Orange, CA 92868, USA; 2 Department of Pediatrics, University of California-Irvine School of Medicine, Orange, CA 92868, USA; 3 Division of Biostatistics, University of Minnesota School of Public Health, Minneapolis, MN 55455, USA; rudser@umn.edu (K.D.R.); jacob004@umn.edu (D.R.J.); 4 School of Kinesiology, University of Minnesota, Minneapolis, MN 55455, USA; denge001@umn.edu; 5 Department of Pediatrics, University of Minnesota Medical School, Minneapolis, MN 55455, USA; braun002@umn.edu (E.A.B.); stein055@umn.edu (J.S.); sinai001@umn.edu (A.R.S.); kelly105@umn.edu (A.S.K.)

**Keywords:** mucopolysaccharidosis, vascular, intima, media, thickness, stiffness, outcome, treatment, lysosomal, structure, function

## Abstract

Treatments for mucopolysaccharidoses (MPSs) have increased longevity, but cardiovascular disease causes mortality in a significant percentage of survivors. Markers must be developed to predict MPS cardiac risk and monitor efficacy of investigational therapies.MPS patients underwent carotid artery ultrasonography from which carotid intima-media thickness (cIMT) and three measures of arterial stiffness were calculated: carotid artery distensibility (cCSD), compliance (cCSC), and incremental elastic modulus (cIEM). MPS carotid measurements were compared to corresponding data from pediatric and adult healthy cohorts. 33 MPS patients (17 MPS I, 9 MPS II, 4 MPS IIIA, and 3 MPS VI; mean age 12.5 ± 4.7 years), 560 pediatric controls (age 13.1 ± 4.0 years), and 554 adult controls (age 39.2 ± 2.2 years) were studied. Age and sex-adjusted aggregate MPS cIMT (0.56 ± 0.05 mm) was significantly greater than both pediatric (+0.12 mm; 95% CI +0.10 to +0.14 mm) and adult (+0.10 mm; 95% CI +0.06 to +0.14 mm) control cohorts; similar findings were observed for all MPS subtypes. Mean MPS cIMT approximated the 80th percentile of the adult cohort cIMT. MPS patients also demonstrated significantly increased adjusted arterial stiffness measurements, evidenced by reduced cCSD, cCSC, and increased cIEM, compared to pediatric and adult control cohorts. Regardless of treatment, MPS patients demonstrate increased cIMT and arterial stiffness compared to healthy pediatric and adult controls. These data suggest that relatively young MPS patients demonstrate a “structural vascular age” of at least 40 years old.

## 1. Introduction

The mucopolysaccharidoses (MPSs) are a group of inborn errors of metabolism linked by a deficiency in one of eleven lysosomal hydrolases that degrade glycosaminoglycans (GAGs), modified glycan components such as heparan, keratan, dermatan, and chondroitin sulfate found in cells and extracellular matrix throughout the body. Lysosomal accumulation of GAGs causes multi-systemic disease, including cognitive impairment of variable severity, hepatosplenomegaly, skeletal dysplasia with degenerative arthritis, cardiac valvular dysplasia, and arterial vascular disease [1]. MPS patients frequently die of cardiovascular disease, which often worsens despite development of treatments such as hematopoietic stem cell transplantation (HSCT) and intravenous enzyme replacement therapy (ERT) [2,3,4,5,6,7,8].

Postmortem analyses have demonstrated arterial luminal stenosis in MPS patients of multiple different types, caused by intimal medial proliferation [9]. We previously utilized non-invasive carotid artery ultrasonography to demonstrate that carotid intima-media thickness (cIMT) [10], an in vivo marker of intimal medial proliferation, in patients with MPSs was significantly thicker than matched controls [11]. We corroborated this finding in a larger, dual-center assessment of 25 MPS Type I and II patients and discovered that our predominantly pediatric cohort of MPS patients demonstrated increased carotid artery stiffness compared to a healthy pediatric control cohort [12].

We report herein additional studies of cIMT and carotid stiffness in an expanded cohort of MPS patients, including novel assessments in individuals with MPS III and MPS VI. MPS III, also known as Sanfilippo syndrome, has classically been associated with neurodegeneration arising from heparan sulfate storage within neurons and astrocytes of the central nervous system. MPS VI, or Maroteaux–Lamy syndrome, is a rare form of MPS that results in dermatan sulfate storage. We additionally highlight the severe abnormalities in arterial structure and function in MPS patients by comparing findings from our cohort to not only a healthy pediatric cohort, but also to a general adult cohort, and propose the underlying pathophysiology of our findings.

## 2. Results

### 2.1. Study Cohort Descriptions

The pediatric control cohort had 560 individuals with ages ranging from 6 to 25 years (mean ± SD, 13.1 ± 4.0 years), with 301 (53.8%) male controls and 259 (46.2%) female controls. The adult control cohort had 554 individuals with ages ranging from 18 to 49 years (mean ± SD, 39.2 ± 2.2 years), with 268 (48.4%) male controls and 285 (51.4%) female controls. The MPS cohort was composed of 33 individuals, ranging in age from 6 to 25 years (mean ± SD, 12.5 ± 4.7 years). Since all enrolled MPS Type II patients were male, the gender ratio of the entire MPS cohort was 2.3:1, male to female. Of the non-MPS II patients, there were 14 males (58.3%) and 10 females (42.7%). Control cohorts were large to encompass sufficient range of ages and measurements to allow for statistical comparisons to the smaller MPS cohort. Additional information regarding these cohorts, including measurements of height, weight, body mass index, and blood pressure, is summarized in Table 1.

Within the MPS cohort of 33 patients, 17 (51.5%) had MPS Type I, 9 (27.3%) had MPS Type II, 4 (12.1%) had MPS Type IIIA, and 3 (9.1%) had MPS Type VI. No patients with MPS IIIB, IIIC, or IIID were enrolled. All but the MPS III patients were, or had, received treatment with HSCT and/or ERT. Additional information regarding basic anthropometric measurements, heart rate, and blood pressure of the MPS cohort, including data from each MPS type, is summarized in Table 2.

### 2.2. Carotid Intima Media Thickness and Stiffness Measurements

The unadjusted carotid measurements from the control and MPS cohorts, including specific measurements from each MPS type, are found in Table 3, while age and sex-adjusted comparisons are summarized in Table 4. The MPS cohort cIMT was 0.56 ± 0.05 mm, which was significantly greater than cIMT of both the pediatric control cohort (0.44 ± 0.04 mm, adjusted *p* < 0.001) and the adult control cohort (0.52 ± 0.09 mm, adjusted *p* < 0.001). MPS cohort cIMT–luminal diameter ratio, which accounts for adaptive responses to increased height and changes in tensile stress, continued to be increased compared to pediatric and adult cohorts. The MPS cohort demonstrated significantly increased carotid stiffness compared to both control cohorts as well, which quantitatively corresponds with lower cCSD, cCSC, and higher cIEM. Following adjustment for age and sex, the MPS cohort had lower cCSD (−4.08%, *p* = 0.001; −4.37%, *p* = 0.035) compared to pediatric and adult control cohorts, respectively, lower cCSC (−0.03 mm^2^/mm Hg, *p* < 0.001; −0.04 mm^2^/mm Hg, *p* = 0.003) compared to pediatric and adult control cohorts, respectively, and higher cIEM (410.83 mm Hg, *p* < 0.001; 501.93 mm Hg, *p* = 0.005). As expected, cIMT and carotid stiffness in the MPS cohort were increased in males and with increasing age.

The mean MPS IIIA cIMT was among the highest (0.59 mm) of the MPS subtypes; MPS IIIA patients also demonstrated elevated mean carotid stiffness as evidenced by lower cCSD (21.59%), cCSC (0.14 mm^2^/mm Hg), and higher cIEM (1210 mm Hg) versus the pediatric cohort (32.03%, 0.16 mm^2^/mm Hg, 951 mm Hg, respectively). The MPS VI patients had some of the more abnormal mean cIMT (0.57 mm), cCSD (25.76%), cCSC (0.15 mm^2^·mm Hg^−1^), and cIEM (1467 mm Hg) measurements of the MPS cohort.

**Figure 1:**
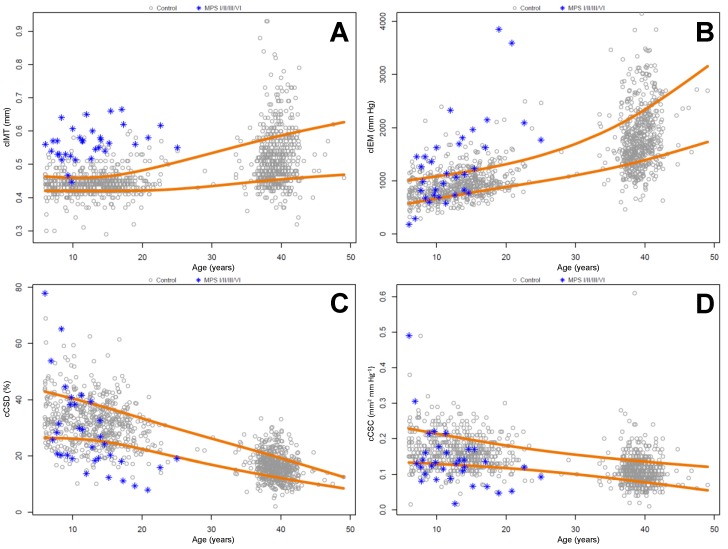
Scatter plots of carotid measurements from MPS cohort (blue stars), pediatric, and adult control cohorts (gray circles) versus age. (**A**) Carotid intima-media thickness; (**B**) carotid incremental elastic modulus; (**C**) carotid cross-sectional distensibility; (**D**) carotid cross-sectional compliance. The orange lines in each panel denote the 20th (lower) and 80th (upper) percentiles of control cohort measurements.

Nearly all (97%, 32/33) patients in the MPS cohort had cIMT measurements that exceeded the 80th percentile of age-matched pediatric controls. Strikingly, 94% (31/33) patients in the MPS cohort had cIMT measurements that were above the 50th percentile of the adult cohort (Table 5). Approximately half of the MPS cohort had stiffness measurements in the lowest quintile (cCSD and cCSC) or highest quintile (cIEM) of the pediatric cohort. A smaller, but still significant portion of the MPS cohort had stiffness measurements below (cCSD and cCSC) or above (cIEM) the adult cohort mean. These results are summarized in Table 5 and Figure 1.

## 3. Discussion

Stem cell transplantation for severe MPS I and enzyme replacement therapies for MPS Types I, II, IVA, and VI are reducing overall disease burden and extending patient lifespans [13]. However, clinical observation, examination of registry data, and large case series indicate that a majority of surviving patients develop progressive cardiovascular disease despite treatment. Cardiac valvular insufficiency, ventricular dysfunction, sudden death, and coronary intimal medial proliferation have all been observed in stably treated patients [14].

We hypothesized that intimal medial proliferation observed in the coronary arteries of deceased MPS patients was occurring throughout the arterial vasculature, including the carotid arteries. This phenomenon would be easily and noninvasively assessed using carotid ultrasonography to measure in vivo carotid intima media thickness and quantify carotid stiffness. Carotid intima media thickness correlates with carotid histology [15] and has been utilized as a validated marker for myocardial infarction and cerebrovascular accidents in other chronic conditions such as diabetes mellitus and hypertension [16].

The cIMT and stiffness parameters of the MPS cohort consistently and positively correlated with male gender and increasing age, which is important because gender and age are known covariates observed from many other community-based studies. In addition, MPS carotid function concordantly (reduced cCSD, cCSC, and increased cIEM) indicated significantly increased stiffness compared to the control pediatric cohort. We previously reported less extensive comparisons in a cohort of MPS I and II patients [12]; in this study, our study cohort was increased by nearly one-third by inclusion of MPS IIIA and VI patients. Our findings establish greater evidence for vascular disease across all MPS types by demonstrating abnormal findings in MPS IIIA and VI patients. The four patients with MPS IIIA, who also had mitral and aortic valve insufficiency, suggest the possibility that Sanfilippo type A, a disorder primarily known for its neurodegenerative manifestations, may also develop significant cardiovascular pathology. Cardiovascular studies that include all four MPS III subtypes must be performed to further substantiate this possibility. If demonstrable throughout the Sanfilippo syndromes, future therapeutics for MPS III must then encompass alleviation of somatic disease in addition to neuropathology.

The extent of MPS vascular pathology is further accentuated by comparison to the adult cohort, whose mean age was 39.2 years. The MPS cohort, whose mean age was much younger at 12.5 years, still demonstrated significantly increased cIMT and carotid stiffness even after adjustment for gender and age. All but two MPS patients had cIMT measurements exceeding the 50th percentile for adult controls, and the mean cIMT of the MPS cohort was at the 80th percentile of the adult cohort. In effect, the structural “vascular age” (as measured by cIMT) of an early adolescent MPS patient is equivalent to that of an approximately 40-year-old control adult.

The four MPS IIIA patients were the only untreated patients in the cohort and yet demonstrated some of the most significant carotid and valvular disease. On the other hand, since all of the MPS Type I, II, and VI patients either had undergone HSCT, or were receiving ERT at the time of assessment, their cIMT and stiffness parameters clearly indicate that current treatments for MPS fail to completely ameliorate the full extent of the abnormalities in either carotid structure or function. Vascular disease therefore represents an additional unmet therapeutic area for MPS patients, together with cardiac valvular insufficiency and stenosis.

Strengths of this study include the relatively large sample size for a rare disorder such as MPS and standardized techniques performed in the same core laboratory. Limitations include the small cohort sizes of each individual MPS type prohibiting meaningful intra-MPS type comparisons. In addition, the cross-sectional design of our study places constraints on some of the conclusions we are able to draw. Longitudinal studies of changes in carotid structural and functional markers will be necessary to adequately assess its clinical utility in regard to identifying the highest risk patients and evaluating the effectiveness of various treatments for MPS.

Although our MPS cohort did not have patients with MPS Types IIIB, IIIC, IIID, IVA, or VII, we hypothesize that all MPS types will demonstrate abnormally increased cIMT and stiffness. Arteries normally contain all types of GAGs [17,18] that accumulate in each of these disorders. This conjecture is further supported by the histopathology observation of myointimal proliferation and fragmented elastin in coronary arteries or aortas of MPS IIIB, IIIC, IVA, and VII patients [9,19,20,21,22]. We are conducting ongoing carotid ultrasonography studies to confirm this hypothesis in MPS IVA patients.

We hypothesize that cIMT is an in vivo marker of arterial GAG storage and intimal medial proliferation in MPS patients. The etiology of increased carotid stiffness in MPS patients is unlikely to be GAG storage alone, but rather by secondary alterations of arterial parenchyma induced by storage. Arterial inflammation, proliferation of myofibroblasts and vascular smooth muscle cells, together with attenuation and fragmentation of elastin fibrils have been observed not only in human MPS, but also in animal models [21,22,23,24]. These sequelae reduce vascular compliance via mechanisms similar to those observed in atherosclerosis [25] and Marfan syndrome [26]. Since supra-normal circulating lysosomal enzymatic levels are required for normalization of aortic pathology in animal models [27,28], therapies targeting MPS cardiovascular disease must focus on maximizing enzymatic uptake in vascular intima/media, or abrogating vascular intraparenchymal inflammation, cellular proliferation, and disruption of elastin laminae. Systemic gene therapy [27] or anti-inflammatory agents [24] show promise in preclinical studies. Human therapeutic trials anticipated in the near future should consider utilizing cIMT and carotid stiffness measurements as potential markers of cardiovascular efficacy.

## 4. Materials and Methods

### 4.1. Human Subjects

This study was approved by the respective institutional review boards at the University of Minnesota and CHOC Children’s Hospital (Study #1202M09721, approved 20 February 2012, renewed 1 October 2015; Study #1201114, approved 28 February 2012, renewed 17 August 2016, respectively). The studies providing data for the pediatric and adult control cohorts were approved by the institutional review board at the University of Minnesota (Study #0608M91586, approved 28 April 2005, renewed 16 September 2016; and #0411M65666, approved 8 March 2007, renewed 24 February 2016, respectively). All studies were conducted according to the Declaration of Helsinki, and written informed consent and verbal assent was provided by all parents and participants, respectively.

### 4.2. Study Design

This study was a dual-center, cross-sectional assessment of carotid imaging data obtained from 33 patients with biochemically and/or molecularly confirmed MPS Types I, II, III, and VI; 560 healthy pediatric control patients; and 554 adult control patients. The pediatric and adult controls were obtained from prior studies of insulin resistance and cardiovascular risk at the University of Minnesota that utilized identical carotid imaging protocols [29,30,31,32]. Clinical data (demographic information, anthropometrics, ethnicity, confirmation of diagnosis, and treatment status) from MPS patients were obtained from chart review.

### 4.3. Carotid Artery Imaging and Analysis

Vascular studies were performed in a quiet environment at constant ambient temperature. Vascular images were obtained with conventional ultrasound scanners with participants in the supine position. De-identified images were acquired and stored on electronic media (Children’s Hospital Orange County scans) or a secured personal computer (University of Minnesota scans) for later analysis by one reader (DRD) at a central laboratory (University of Minnesota) using an electronic wall-tracking software program (Vascular Research Tools 5, Medical Imaging Application, LLC, Coralville, IA, USA).

Following at least 10 min of quiet rest in the supine position, vascular images were obtained from the left common carotid artery with a linear array probe held at a constant distance from the skin and at a fixed point over the imaged artery, approximately 1 cm proximal from the carotid bifurcation bulb. Depth and gain settings were set to optimize images of the lumen/arterial wall interface. Images were collected at 20 frames per second for 10 s (200 frames) to ensure the capture of full arterial diameter change during a cardiac cycle. Carotid intima media thickness (cIMT) was measured from the far wall of the left common carotid. The mean diameter through the 10 s cycle was used to calculate carotid compliance (cCSC) and distensibility (cCSD) as per Wang et al. [12]. Systolic and diastolic blood pressures were recorded with an automated blood pressure sphygmomanometer during the 10 s carotid measurements and utilized to calculate incremental elastic modulus (cIEM). As vessel stiffness increases, cCSD and cCSC decline while cIEM increases; rising vessel elasticity has an opposite effect with increased cCSD and cCSC and reduced cIEM.

### 4.4. Statistical Analysis

Descriptive statistics were tabulated separately for the healthy control and MPS groups, which included the mean and standard deviation for continuous variables and frequency for categorical variables. Quantile curves were based on quantile regression using the method of Portnoy [33] with natural cubic splines on age with 4 degrees of freedom. Age- and gender-adjusted results and associations of stiffness measures with cIMT used linear regression and the *t*-distribution with corresponding model degrees of freedom for confidence intervals and *p*-values. All analyses were conducted using R v3.2.4 (R Foundation for Statistical Computing, Vienna, Austria) [34].

## 5. Conclusions

Using noninvasive vascular ultrasonography, we demonstrate significantly increased carotid intima media thickness and stiffness in patients with mucopolysaccharidoses Types I, II, IIIA, and VI when compared with healthy pediatric and adult controls. As measured by carotid intima media thickness, the mean structural “vascular age” of studied MPS patients was more than triple that of their mean chronologic age. Carotid abnormalities were present in patients with MPS IIIA, a condition considered to manifest with predominantly neurodegenerative symptoms, as well as treated MPS patients. Measures of carotid artery structure and function may prove useful in tracking outcomes in the MPS population and might have utility as a measure of treatment efficacy in future clinical trials in this group.

## Figures and Tables

**Table 1:** Descriptive cohort characteristics: values presented are mean (standard deviation) or *n* (%) where indicated. Superscripts denote number of individuals (if any) with missing data; abbreviations: BMI: body mass index; SBP: systolic blood pressure; DBP: diastolic blood pressure; HR: heart rate; n.a.: not applicable; n.m.: not measured.

Covariate	Control: Pediatric	Control: Adult	MPS
	(*n* = 560)	(*n* = 554)	(*n* = 33)
Female	259 (46.2%)	285 (51.4%) ^1^	10 (30.3%)
Age at visit (years)	13.14 (4.01)	39.16 (2.24) ^1^	12.47 (4.66)
Height (cm)	155 (18.30)	171 (12.80) ^110^	132 (16.59)
Weight (kg)	53.80 (23.54)	86.39 (23.33) ^108^	36.66 (16.74)
BMI (kg/m^2^)	21.44 (5.84)	29.46 (7.47) ^114^	20.03 (4.50)
BMI percentile	63.44 (27.33)	n.a.	65.89 (26.29)
SBP (mm Hg)	106 (10.42) ^4^	125 (15.59) ^3^	106 (10.98)
DBP (mm Hg)	58.00 (7.78) ^4^	71.97 (10.39) ^3^	55.00 (13.73)
HR (min^−1^)	79.39 (72.38) ^5^	n.m.	87.70 (14.54)
**Race**			
White	379 (67.7%)	371 (67.0%)	27 (81.8%)
Black	124 (22.1%)	138 (24.9%)	2 (6.1%)
Native American	14 (2.5%)	16 (2.9%)	0 (0.0%)
Asian	16 (2.9%)	18 (3.2%)	0 (0.0%)
Hispanic	3 (0.5%)	1 (0.2%)	0 (0.0%)
Other	24 (4.3%)	1 (0.2%)	3 (9.1%)
Missing race	0 (0.0%)	9 (1.6%)	1 (3.0%)

**Table 2:** Descriptive characteristics of mucopolysaccharidosis (MPS) types: values presented are mean (standard deviation) or *n* (%) where indicated. Superscripts denote number of individuals (if any) with missing data. For the MPS cohort, missing values are due to age of participant being beyond BMI percentile tables, which are only available for pediatrics.

Covariate	MPS	MPS I	MPS II	MPS IIIA	MPS VI
	(*n* = 33)	(*n* = 17)	(*n* = 9)	(*n* = 4)	(*n* = 3)
Male	23 (69.7%)	10 (58.8%)	9 (100.0%)	3 (75.0%)	1 (33.3%)
Female	10 (30.3%)	7 (41.2%)	0 (0.0%)	1 (25.0%)	2 (66.7%)
Treatment: HSCT only	9 (27.3%)	9 (52.9%)	0 (0.0%)	0 (0.0%)	0 (0.0%)
Treatment: HSCT, now ERT	3 (9.1%)	2 (11.8%)	0 (0.0%)	0 (0.0%)	1 (33.3%)
Treatment: ERT only	17 (51.5%)	6 (35.3%)	9 (100.0%)	0 (0.0%)	2 (66.7%)
Treatment: None	4 (12.1%)	0 (0.0%)	0 (0.0%)	4 (100.0%)	0 (0.0%)
Age at visit (years)	12.47 (4.66)	12.18 (4.72)	10.80 (3.17)	16.97 (5.48)	13.13 (5.50)
SBP (mm Hg)	106 (10.98)	106 (10.99)	103 (11.96)	118 (5.91)	103 (2.65)
DBP (mm Hg)	55.00 (13.73)	50.18 (13.74)	54.11 (6.94)	73.00 (15.08)	61.00 (8.19)
HR (min^−1^)	87.70 (14.54)	87.06 (16.05)	92.00 (12.30)	81.75 (17.63)	86.33 (9.61)
Height (cm)	132 (16.59)	133 (15.59)	134 (11.48)	136 (26.78)	115 (19.04)
Weight (kg)	36.66 (16.74)	37.02 (18.61)	38.05 (12.65)	40.77 (22.30)	24.97 (8.82)
BMI (kg/m^2^)	20.03 (4.50)	19.82 (5.46)	20.54 (3.41)	20.93 (4.37)	18.43 (2.06)
BMI percentile	65.89 (26.29) ^4^	60.28 (24.62) ^3^	81.63 (14.21)	65.67 (39.58) ^1^	45.07 (37.07)
**Race**					
White	27 (81.8%)	15 (88.2%)	6 (66.7%)	4 (100.0%)	2 (66.7%)
Black	2 (6.1%)	0 (0.0%)	2 (22.2%)	0 (0.0%)	0 (0.0%)
Native American	0 (0.0%)	0 (0.0%)	0 (0.0%)	0 (0.0%)	0 (0.0%)
Asian	0 (0.0%)	0 (0.0%)	0 (0.0%)	0 (0.0%)	0 (0.0%)
Hispanic	0 (0.0%)	0 (0.0%)	0 (0.0%)	0 (0.0%)	0 (0.0%)
Other	3 (9.1%)	2 (11.8%)	1 (11.1%)	0 (0.0%)	0 (0.0%)
Missing race	1 (3.0%)	0 (0.0%)	0 (0.0%)	0 (0.0%)	1 (33.3%)

**Table 3:** Unadjusted mean (SD) carotid measurements of control and MPS cohorts. Measurements of the MPS cohort are further subdivided according to MPS type. Superscripts denote number (if any) of individuals with missing measurements. Abbreviations: cIMT: carotid intima-media thickness; cCSD: carotid cross-sectional distensibility; cCSC: carotid cross-sectional compliance; cIEM: carotid incremental elastic modulus.

Vascular Measure	Control (Pediatric)	Control (Adult)	MPS	MPS I	MPS II	MPS IIIA	MPS VI
	(*n* = 560)	(*n* = 554)	(*n* = 33)	(*n* = 17)	(*n* = 9)	(*n* = 4)	(*n* = 3)
cIMT (mm)	0.44 (0.04)	0.52 (0.09)	0.56 (0.05)	0.57 (0.04)	0.55 (0.07)	0.59 (0.05)	0.57 (0.04)
cCSD (%)	32.03 (8.35)	16.33 (4.73) ^1^	28.36 (15.70)	28.47 (14.30)	32.01 (19.66)	21.59 (2.39)	25.76 (24.28)
cCSC (mm^2^·mm Hg^−1^)	0.16 (0.05)	0.11 (0.04) ^2^	0.14 (0.08)	0.12 (0.06)	0.18 (0.12)	0.14 (0.04)	0.15 (0.14)
cIEM (mm Hg)	951 (379.84)	1856 (755.3) ^2^	1341 (817.06)	1422 (973.19)	1203 (632.77)	1210 (416.98)	1467 (1021.21)

**Table 4:** Comparisons between carotid measures in control and MPS cohorts adjusted for sex and age. Statistically significant comparisons are highlighted in bold. Increasing carotid stiffness corresponds with lower cCSD, cCSC, and higher cIEM, while increasing carotid elasticity corresponds with higher cCSD, cCSC, and lower cIEM.

Vascular Measure	Covariate	Adjusted Difference (95% CI)	*p*-Value
cIMT (mm)	Control (adult) vs. MPS	−0.10 (−0.14, −0.06)	**<0.001**
	Control (pediatric) vs. MPS	−0.12 (−0.14, −0.10)	**<0.001**
	Female	−0.01 (−0.02, −0.01)	**<0.001**
	Age (per 10 years)	+0.02 (0.01, 0.03)	**<0.001**
cCSD (%)	Control (adult) vs. MPS	+4.37 (0.31, 8.42)	**0.035**
	Control (pediatric) vs. MPS	+4.08 (1.64, 6.51)	**0.001**
	Female	+0.02 (−0.79, 0.82)	0.963
	Age (per 10 years)	−6.15 (−7.36, −4.93)	**<0.001**
cCSC (mm^2^·mm Hg^−1^)	Control (adult) vs. MPS	+0.04 (0.01, 0.07)	**0.003**
	Control (pediatric) vs. MPS	+0.03 (0.01, 0.04)	**<0.001**
	Female	−0.01 (−0.02, −0.01)	**<0.001**
	Age (per 10 years)	−0.03 (−0.03, −0.02)	**<0.001**
cIEM (mm Hg)	Control (adult) vs. MPS	−501.93 (−848.54, −155.32)	**0.005**
	Control (pediatric) vs. MPS	−410.83 (−618.72, −202.93)	**<0.001**
	Female	−26.26 (−95.05, 42.52)	0.454
	Age (per 10 years)	+383.35 (279.51, 487.19)	**<0.001**

**Table 5:** Number (%) of MPS patients whose carotid measurements exceeded (in the case of cIMT and cIEM) the 80th percentile of the pediatric control cohort and 50th percentile of the adult control cohort, or were less than (in the case of cCSD and cCSC) the 20th percentile of the pediatric control cohort and 50th percentile of the adult control cohort.

**Vascular Measure**	**Number (%) > Pediatric Cohort 80th Percentile**	**Number (%) > Adult Cohort 50th Percentile**
cIMT (mm)	32 (97%)	31 (94%)
cIEM (mm Hg)	16 (48%)	8 (24%)
**Vascular Measure**	**Number (%) < Pediatric Cohort 20th percentile**	**Number (%) < Adult Cohort 50th percentile**
cCSD (%)	17 (52%)	6 (18%)
cCSC (mm^2^·mm Hg^−1^)	16 (48%)	11 (33%)
